# Tiotropium in asthma: back to the future of anticholinergic treatment

**DOI:** 10.1186/s12948-017-0076-1

**Published:** 2017-12-04

**Authors:** Matteo Bonini, Nicola Scichilone

**Affiliations:** 1 Airways Division, Airways Disease Section, National Heart and Lung Institute (NHLI), Royal Brompton Hospital & Imperial College, Dovehouse Street, London, SW3 6LY UK; 2 Department of Biomedicine and Internal and Specialistic Medicine (DIBIMIS), University of Palermo, Palermo, Italy

**Keywords:** Anticholinergic, Antimuscarinic, Asthma, Bronchodilation, Control, Endotype, Exacerbation, Phenotype, Tiotropium

## Abstract

Asthma is among the most common chronic diseases worldwide; however, despite progresses in the understanding of the patho-physiological mechanisms and advances in the development of new therapeutic options and strategies, the disease remains uncontrolled in a not trivial proportion of subjects. Thus, the need of new molecules to treat the underlying biological and functional abnormalities and to control symptoms is strongly advocated by clinicians. In this scenario, the most recent GINA guidelines have included the use of tiotropium bromide in the most severe and uncontrolled forms of the disease, in addition to treatment with inhaled corticosteroid plus long acting beta adrenergic agents. Indeed, a large body of evidence has accumulated to support the use of tiotropium bromide in asthma. The current review paper provides a state of the art systematic revision of findings on the efficacy and safety of tiotropium in the adult and paediatric asthma population. To this aim, electronic searches were undertaken in the most common scientific databases from the date of inception to March 2017. Robust and high quality evidence showed that tiotropium is effective and safe in both adults and children/adolescents. Predictive markers of response have been also suggested, as well as cost–benefit analyses reported. The tiotropium bronchodilator effect seems to be not solely related to the reduction of the smooth muscle tone. However, the observations on anti-inflammatory properties or reduction in mucus production, despite highly interesting, have been only demonstrated in in vitro studies and animal models, therefore advocating for further specifically designed investigations.

## Background

Asthma is a major health concern worldwide, with a global prevalence of approximately 300 million, and an estimation of increasing figures up to 400 million people worldwide by 2025 [1]. Asthma is characterized by airway inflammation, reversible airway obstruction, and airway hyperresponsiveness which lead to respiratory symptoms that vary in terms of frequency and severity. Despite treatment per management guidelines [2], a vast proportion of patients experiences uncontrolled forms of the disease [3], thus representing a relevant unmet medical need. Indeed, uncontrolled asthma is responsible for impaired quality of life, increased number of visits to the emergency room and hospitalizations and disproportionate use of healthcare resources [4].

The observed variability in clinical response to currently available therapies has been related to distinctive asthma phenotypes and endotypes [5–8]. However, the evidence of poor control of symptoms despite novel and more targeted treatments [9] has led to explore novel treatment strategies. In this respect, anticholinergic drugs are being considered an alternative bronchodilator therapeutic option to beta-2 agonists for asthma. Beta-2 adrenergic drugs are the mainstay of asthma management and are the most commonly adopted treatment for preventing and reversing bronchial obstruction [10], however high heterogeneity in individual responses, occurrence of tolerance and side-effects have been reported with their use [11–13]. It is known that ancient Ayurvedic medicine already used *Datura stramonium* (a plant with anticholinergic effects) for asthma treatment. Subsequently, the discovery of atropine, a potent competitive inhibitor of acetylcholine at postganglionic muscarinic receptors, and more importantly the demonstration of the importance of the parasympathetic nervous system in bronchoconstriction, renewed interest on the potential value of antimuscarinic agents in asthma [10]. Vagal innervation is in fact currently considered the major determinant of airway tone and represents the reversible component of airflow obstruction [14]. An up-regulated release of acetylcholine (ACh) causes an increased bronchial tone, bronchial hyperresponsiveness and reflex bronchoconstriction, thus leading to the narrowing of the airways. Bronchoconstriction is primarily regulated by five muscarinic receptors (M_R_); M_1_, M_2_ and M_3_ are expressed in the lung and in the bronchial tree. M_1R_ are mainly distributed in the peripheral lung tissue and in the alveolar walls within parasympathetic ganglia and regulate cholinergic transmission. M_2R_ are found in post-ganglionic nerves where they serve as auto-receptors, on smooth muscle cells (SM) and on fibroblasts. M_3R_ are predominantly expressed in SM cells and mediate SM ACh-induced contraction. In central airways, SM contraction is mediated by vagal innervation, whereas in the peripheral airways the function of M_3R_ is mediated by ACh released in response to inflammatory stimuli. M_3R_ can also be found in sub-mucosal glands where they are responsible for mucus secretion. Among the short-acting anti-cholinergic molecules, ipratropium bromide and oxitropium bromide have long been adopted as asthma relievers. Furthermore, a large body of evidence has accumulated in recent years to support the use of the long-acting anti-muscarinic tiotropium bromide in asthma [15, 16], and its use is now recommended by international guidelines (GINA 2016) for chronic treatment of adult patients with most severe and frequently exacerbated asthma [2], in addition to inhaled corticosteroids (ICS) in combination with long-acting beta-2 agonists (LABA). It has been also suggested that tiotropium exerts its beneficial effects through mechanisms other than the reduction on the cholinergic tone of the airways, some of which still need to be confirmed in humans [17].

The current paper aims at providing a state of the art systematic review of efficacy and safety of tiotropium in asthma. To this scope, the main findings on the role of tiotropium in pediatric and adult asthmatic populations were selected in a rigorous and unbiased manner, and reported in light of the current pharmacological treatment recommendations for asthma.

## Methods

Electronic searches were undertaken in MEDLINE, Web of Science, the Cochrane Library and Scopus databases. The registers were searched using the keywords “asthma” AND “tiotropium” from the date of inception to March 2017. Following the removal of duplicates, the authors independently selected papers of potential interest on the basis of titles and abstracts for a full-text assessment and reached an agreement in cases of lack of consensus. Original studies of any design (except for single case reports due to low quality), published in English, performed in humans and primarily addressing the efficacy and/or safety of tiotropium in asthma were considered eligible for being included in the present review. In addition, reference lists of included manuscripts, recent reviews and textbooks were hand-searched for further relevant citations.

## Results

**Fig. 1:**
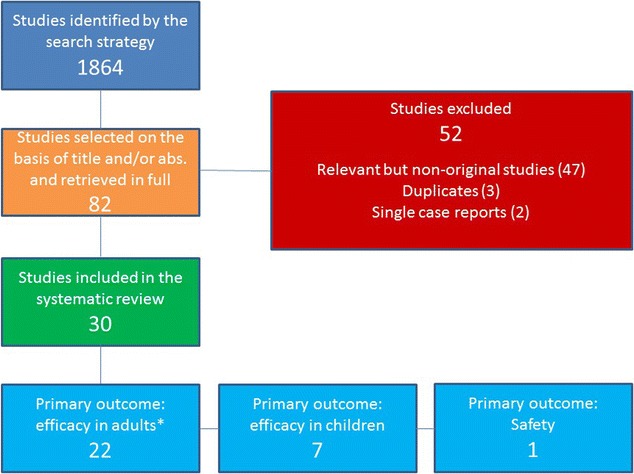
Search-strategy flow-chart. *Including 5 studies on predictors of response and 1 study on cost-effectiveness

The search strategy yielded 1864 articles (MEDLINE 211, Web of Science 420, the Cochrane Library 229 and Scopus 1004). Among these, 30 studies met the criteria for being included in the review and are reported below according to their primary aim (i.e. efficacy or safety). In regards to efficacy further distinctions have been also made depending on the target study population (i.e. adults or children/adolescents), the type of the effects investigated (bronchodilator or non-bronchodilator) and the findings on predictive markers of response and cost–benefit (Fig. 1).

### Bronchodilator effects in adults

A proof of concept double blind, randomised, placebo-controlled, crossover study aiming to evaluate the effects of halving ICS dosage adding salmeterol, or salmeterol plus tiotropium was conducted in 2007 [18]. Eighteen non-smoking severe asthmatics were run-in for 4 weeks on HFA-fluticasone propionate 1000 mcg daily, and were then randomised to 4 weeks of either (a) HFA-fluticasone propionate 500 mcg twice a day + salmeterol 100 mcg twice a day + HFA-tiotropium bromide 18 mcg once a day; or (b) fluticasone propionate 500 mcg twice a day + salmeterol 100 mcg twice a day + matched placebo. Spirometry and body plethysmography were performed. Adding salmeterol to half the dose of fluticasone led to a significant improvement vs. baseline in the morning peak expiratory flow (PEF) and airway resistance (RAW). The combination salmeterol/tiotropium produced similar improvements in PEF and RAW, but also significantly improved the forced expiratory volume in the first second (FEV1) by 0.17 l (CI 0.01–0.32 l), FVC 0.24 l (CI 0.05–0.43 l) and reduced fraction exhaled NO (FeNO) by 2.86 ppb (CI 0.12–5.6 ppb).

In order to assess if there was any difference among the protective effect of ipratropium, oxitropium and tiotropium against methacholine-induced bronchoconstriction, 44 patients with intermittent asthma and a PD20 FEV1 < 200 mcg were selected [19]. At baseline, they had a mean FEV1% predicted of 98.8 ± 8.5 and mean PD15 FEV1 of 111.8 ± 61.0 mcg. After 72 h, all patients underwent a second methacholine challenge being given ipratropium (40 µg by MDI; n = 14), oxitropium (200 µg by MDI; n = 14) or tiotropium (18 µg by Handihaler; n = 16) 60 min before the test. The FEV1% increase was significantly higher in the oxitropium (6.7 ± 4.8%) and tiotropium groups (6.1 ± 2.5%) compared to the ipratropium group (3.8 ± 1.9%). Furthermore, after oxitropium and tiotropium, the PD15 (1628 ± 955.7 and 1595.5 ± 990 µg, respectively) was significantly higher in comparison to that following ipratropium (532.2 ± 434.8 µg).

In a three-way, double-blind, triple-dummy crossover trial involving 210 patients with inadequately controlled asthma, the addition of tiotropium to ICS, as compared with a doubling of the dose of the ICS (primary superiority comparison) or the addition of the LABA salmeterol (secondary non-inferiority comparison) was evaluated [20]. The use of tiotropium resulted superior in the primary outcome, when compared with a doubling of the dose of ICS, as assessed by measuring PEF, with a mean difference of 25.8 l/min (p < 0.001) and in most secondary outcomes (i.e. evening PEF, the proportion of asthma control days, FEV1 before bronchodilation and daily symptom scores). The addition of tiotropium was also non-inferior to the addition of salmeterol for all assessed outcomes and increased the pre-bronchodilator FEV1 more than what did salmeterol, with a difference of 0.11 l (p = 0.003).

Kerstjens et al. sought to compare through a randomized, double-blind, crossover study with three 8-week treatment periods, the efficacy and safety of 2 doses of tiotropium (5 and 10 mcg daily) administered through the Respimat inhaler compared with placebo as add-on therapy in patients with uncontrolled severe asthma, despite maintenance treatment with at least a high-dose ICS plus a LABA [21]. The primary endpoint was the peak FEV1 at the end of each treatment period. In the 107 adult patients enrolled, the peak FEV1 was significantly higher with 5 mcg (difference: 139 ml; 95% CI 96–181 ml) and 10 mcg (difference: 170 ml; 95% CI 128–213 ml) of tiotropium than with placebo (both p < 0.0001). No significant difference was observed between the active doses. Trough FEV1 and daily home PEF measurements at the end of the dosing interval were also higher with tiotropium at both doses. Adverse events (AE) were balanced across groups except for dry mouth, which was more common on tiotropium 10 mcg.

In two replicate, randomized, controlled trials involving 912 patients with asthma who were receiving ICS and LABA, authors compared the effect on lung function and exacerbations of adding tiotropium (a total dose of 5 μg) or placebo, both delivered by a soft-mist inhaler once daily for 48 weeks [22]. All the patients were symptomatic, had a post-bronchodilator FEV1 ≤ 80% of predicted and a history of at least one severe exacerbation in the previous year. At 24 weeks, the mean (± SE) change in the peak FEV1 from baseline was significantly greater with tiotropium than with placebo in the two trials: a difference of 86 ± 34 ml in trial 1 (p = 0.01) and 154 ± 32 ml in trial 2 (p < 0.001). The trough FEV1 also significantly improved in both trials with tiotropium, as compared with placebo. Furthermore, the addition of tiotropium increased the time to the first severe exacerbation (282 days vs. 226 days), with an overall reduction of 21% in the risk of a severe exacerbation (hazard ratio, 0.79; p = 0.03). AE were similar in the two groups and no deaths occurred.

The effects of a tiotropium single-dose on lung function were investigated in 18 severe asthmatics with and without emphysematous changes despite maximal recommended treatments with high-dose ICS and inhaled LABA through a double-blind, placebo-controlled, crossover study [23]. The primary efficacy outcome was the relative change in FEV1 from baseline to 60 min. Subsequently, the patients were treated with tiotropium inhaled once daily for 12 weeks in an open label manner, and lung function and symptoms were evaluated. At baseline, patients with emphysema had a mean FEV1% predicted of 55.9% before tiotropium and 56.8% before placebo, while in those without emphysema this was 77.4 and 77.6% respectively. In the first group, the increase from baseline FEV1 was 12.6% higher after tiotropium than placebo, while in the second the improvement was 5.4% higher after tiotropium than placebo. Chronic tiotropium administration also resulted in improved lung function and symptoms, particularly in asthmatics with emphysema.

To further investigate the efficacy and safety of three different doses of tiotropium Respimat as add-on to ICS in symptomatic patients with moderate persistent asthma, a randomised, double-blind, placebo-controlled, four-way crossover study, was conducted [24]. Patients were randomised to tiotropium Respimat 5, 2.5, 1.25 μg or placebo, once daily in the evening. Each treatment was administered for 4 weeks, without washout between treatment periods. Eligibility criteria included FEV1 60–90% of predicted and a seven-question Asthma Control Questionnaire (ACQ-7) score ≥ 1.5. Patients were required to continue maintenance treatment with stable medium-dose ICS for at least 4 weeks prior to and during the treatment period. LABA were not permitted during the treatment phase. The primary efficacy endpoint was peak FEV1 measured within 3 h after dosing (peak FEV1 0–3 h) at the end of each 4-week period, analysed as a response (change from baseline). In total, 149 patients were randomised and 141 completed the study. Statistically significant improvements in peak FEV1 0–3 h response were observed with each tiotropium Respimat dose versus placebo (all p < 0.0001) being the largest with tiotropium 5 μg (188 ml). Trough FEV1 and FEV1 area under the curve (AUC) responses were also significantly greater with each tiotropium dose than with placebo (all p < 0.0001). Occurrence of AE was comparable between placebo and all tiotropium Respimat groups.

Medical records of adults with asthma who were prescribed tiotropium were obtained from the UK Optimum Patient Care Research Database for the period 2001–2013 [25]. Two primary outcomes were compared in the year before (baseline) and the year after (outcome) addition of tiotropium: exacerbations (i.e. asthma-related hospital emergency department attendance, inpatient admission, or acute oral corticosteroid course) and acute respiratory events (i.e. exacerbation or antibiotic prescription with lower respiratory consultation). Secondary outcomes included lung function tests and short-acting β2 agonist (SABA) usage. Comparing baseline and outcome years of the 2042 study patients, the percentage of patients having at least one exacerbation decreased from 37 to 27% (p = 0.001) and the percentage of those having at least one acute respiratory event decreased from 58 to 47% (p = 0.001).

To investigate whether the dosing regimen of tiotropium, delivered via the Respimat SoftMist inhaler, affected 24 h bronchodilator efficacy and safety in asthmatic patients who were symptomatic despite medium-dose ICS, a randomised, double-blind, placebo-controlled, crossover study with 4-week treatment periods of tiotropium 5 mcg (once-daily) and 2.5 mcg (twice-daily) was performed [26]. The primary efficacy endpoint was FEV1 AUC from 0 to 24 h (0–24 h) at the end of each treatment period. Secondary endpoints included peak FEV1 0–24 h, trough FEV1, morning and evening PEF and pharmacokinetic assessments. Ninety-four patients were randomised and 89 (94.7%) completed the study. Significant and comparable bronchodilation was achieved over a 24-h period with both tiotropium dosing regimens. FEV1 AUC 0–24 h response (mean ± SE) was significantly greater with both tiotropium dosing regimens (once-daily 5 mcg: 158 ± 24 ml; twice-daily 2.5 mcg; 149 ± 24 ml; both p < 0.01) when compared with placebo. Improvements in peak FEV1 0–24 h, trough FEV1 and pre-dose a.m./p.m. PEF with both dosing regimens versus placebo were also statistically significant, with no difference between the tiotropium treatment regimens. Total systemic exposure and tolerability were comparable between study arms.

The study of Rajanandh and coworkers aimed to compare the 6 month efficacy and safety of formoterol (12 mcg), montelukast (10 mg), doxofylline (400 mg), or tiotropium (18 mcg) in combination with a low-dose budesonide (400 mcg) in patients with mild to moderate persistent asthma [27]. Outcomes included FEV1, Saint George Respiratory Questionnaire (SGRQ) scores, asthma symptom scores (daytime and night time), assessment of tolerability and rescue medication use. A total of 297 patients completed the study. In all 4 groups, significant improvements were observed in all the outcome measures, with formoterol treatment having greater and earlier improvements than the other 3 add-on medications. No patients discontinued the treatment because of AE.

Two 24-week, replicate, randomised, double-blind, placebo-controlled, parallel-group, active comparator trials were performed at 233 sites in 14 countries [28]. Eligible patients were aged 18–75 years with symptomatic asthma and a pre-bronchodilator FEV1 of 60–90% of predicted despite use of medium-dose ICS and had never smoked or were ex-smokers for ≥ 1 year with ≤ 10 pack-years. Patients were randomly assigned (1:1:1:1), with computer-generated pseudorandom numbers, to receive once-daily tiotropium 2.5 or 5 μg, twice-daily salmeterol 50 μg, or placebo, while maintaining ICS. Pre-specified co-primary endpoints, assessed at week 24, were peak FEV1 response, measured within the first 3 h after evening dosing; trough FEV1 response; and responder rate assessed according to the ACQ-7. Among the 2103 patients enrolled, 519 were randomly assigned to tiotropium 5 μg, 520 to tiotropium 2.5 μg group, 541 to salmeterol and 523 to placebo. A total of 1972 patients (94%) completed the study. Peak and trough FEV1 responses were significantly greater with tiotropium and salmeterol than with placebo and were similar in both studies. With pooled data, difference versus placebo in peak FEV1 was 185 ml (95% CI 146–223) in the tiotropium 5 μg group, 223 ml (95% CI 185–262) in the tiotropium 2.5 μg group, and 196 ml (95% CI 158–234) in the salmeterol group (all p < 0.0001); difference in trough FEV1 was 146 ml (95% CI 105–188), 180 ml (95% CI 138–221), and 114 ml (95% CI 73–155) respectively (all p < 0.0001). There were more ACQ-7 responders in the tiotropium 5 μg (OR 1.32, 95% CI 1.02–1.71; p = 0.035) and 2.5 μg (1.33, 1.03–1.72; p = 0.031) groups, and the salmeterol group (1.46, 1.13–1.89; p = 0.0039), than in the placebo group. Forty-eight patients had serious AE with no difference among the four arms (tiotropium 5 μg n = 11, tiotropium 2.5 μg n = 12, salmeterol n = 11, placebo n = 14).

The primary objective of the study performed by Paggiaro et al. was to evaluate the efficacy of once-daily tiotropium Respimat, compared to placebo, as add-on therapy to low- to medium-dose ICS in adults with symptomatic asthma [29]. A phase III, double-blind, placebo-controlled trial was conducted. Adults with symptomatic asthma (n = 464) receiving 200–400 mcg of budesonide or equivalent doses and a with pre-bronchodilator FEV1 of 60–90% of predicted normal values were randomized to 12 weeks of treatment with once-daily tiotropium Respimat 5, 2.5 mcg, or placebo, as add-on therapy. The primary endpoint was peak FEV1 (0–3 h) response. After 12 weeks, both tiotropium doses were superior to placebo (adjusted mean difference from placebo: 5 mcg = 128 ml; 2.5 mcg = 159 ml (both p < 0.001). Both doses of tiotropium were also superior to placebo with regard to the secondary endpoints (i.e. trough FEV1, FEV1 area under the curve, morning and evening PEF). At last, AE were comparable across the treatment groups.

A Phase II, randomized, double-blind, two-way crossover study comparing two daily dosing regimens of tiotropium for 4 weeks, once-daily 5 mcg (evening dosing) or twice-daily 2.5 mcg (morning and evening dosing), as add-on to maintenance therapy with ICS (400–800 mcg budesonide or equivalent) as controller medication, was conducted to confirm the 24-h bronchodilator efficacy and pharmacokinetic profile of tiotropium Respimat in adults with symptomatic asthma [30]. There was no washout between treatment periods. An increase in the area under the curve of the 24-h FEV1 profile from baseline was observed following once-daily tiotropium 5 mcg (217 ml) and twice-daily 2.5 mcg (219 ml), with no difference between the two regimens. In a subset of the study population, total tiotropium exposure, expressed as area under the plasma concentration versus time curve over 24 h, was comparable between dosing regimens. Unexpected tiotropium plasma levels were observed in two patients, possibly because of contamination.

The aim of the retrospective analysis performed by Abadoglu and Berkto was to assess the effectiveness of tiotropium as an add-on therapy to the standard treatment with high-dose ICS/LABA on asthma control and lung function in patients with severe asthma poorly controlled [31]. Of the 633 asthmatics, 64 (10.1%) patients who were tiotropium add-on treated at least for 3 months were evaluated. Number of exacerbations, emergency department visits, hospitalizations and lung functions of patients belonging to 12 months before starting add-on treatment were compared with those of 12 months after starting add-on treatment. The mean duration of add-on tiotropium treatment was 8.3 ± 0.5 months. Tiotropium improved asthma control in 42.2% of patients and decreased the number of emergency department visits and hospitalizations in 46.9 and 50.0% of them, respectively. While at baseline mean FEV1 and forced vital capacity (FVC) were 57.5 ± 1.9 and 74.3 ± 15.6% respectively, after 12 months of add-on tiotropium these rates increased to 65.5 ± 1.9 and 82.5 ± 15.1%.

A recent study aimed at assessing the effect of tiotropium on airway geometry and inflammation in patients with asthma who were symptomatic despite treatment with ICS/LABA [32]. A total of 53 patients with symptomatic asthma, who received ICS-LABA and who had a pre-bronchodilator FEV1 of 60–90% of the predicted value were randomized to the addition of tiotropium 5 mcg once daily (n = 25) or no add-on (n = 28) to maintenance therapy for 48 weeks. Quantitative computed tomography, FeNO and pulmonary function were measured. Compared to maintenance therapy, the addition of tiotropium significantly decreased airway wall area (WA) and wall thickness (T) corrected for the body surface area (BSA), and improved airflow obstruction. Changes in WA/BSA and T/BSA were significantly correlated with the change in predicted FEV1 (r = − 0.87, p < 0.001, and r = − 0.82, p < 0.001, respectively). No significant difference in the change of FeNO was instead observed between the two treatment groups.

At last, the effects of an inhaled single dose of tiotropium on lung function were investigated through a double-blind, placebo-controlled, crossover study in 9 smoking asthmatics and in 9 who have never smoked, all being treated with ICS and other asthma controllers [33]. Lung function was measured at baseline and at 1, 3, and 24 h after inhalation of 18 mcg of tiotropium or placebo. The primary outcome was a change in FEV1 from baseline. Tiotropium resulted in improved lung function and symptoms both in current smoker and non-smoker asthmatics.

### Bronchodilator effects in children and adolescents

In a case series of 71 paediatric patients, tiotropium was shown to be beneficial in 3 distinct subgroups: as add-on therapy to asthmatics on maximal maintenance medication, as an alternative to high-dose ICS in patients experiencing significant side effects, and in subjects with chronic productive cough as predominant symptom [34]. Interestingly, almost half of the patients who started tiotropium were able to decrease the dose of ICS or stop the use of LABA.

The efficacy and safety of three doses of tiotropium (5, 2.5 and 1.25 μg), administered once-daily via Respimat SoftMist inhaler, were investigated compared to placebo in 139 asthmatic adolescents, symptomatic despite ICS treatment [35]. The change in peak FEV1 within 3 h post-dose from baseline (peak FEV1 0–3 h) was chosen as primary efficacy endpoint and resulted significantly improved in subjects who were administered tiotropium 5 μg. Overall incidence of AE, for the majority mild to moderate, was balanced across treatment groups, with no dose dependent observations.

In a similar Phase II, double-blind, placebo-controlled, incomplete-crossover, dose-ranging study, children aged 6–11 years with symptomatic asthma were randomised to receive once-daily tiotropium Respimat 5, 2.5, 1.25 μg or placebo, add-on to medium-dose ICS with or without a leukotriene modifier, during a 12-week treatment period [36]. For the primary end point (peak FEV1 0–3 h), the adjusted mean responses with tiotropium Respimat 5 μg (272 ml), 2.5 μg (290 ml) and 1.25 μg (261 ml) were all significantly greater than with placebo (185 ml; p = 0.0002, p < 0.0001 and p = 0.0011, respectively). Furthermore, the safety and tolerability of all doses of tiotropium were comparable with those of placebo, with no serious side effects and no events leading to discontinuation.

Eighty children with newly diagnosed moderate persistent asthma were randomly assigned to fluticasone propionate aerosol or fluticasone propionate aerosol plus tiotropium for 12 weeks [37]. Lung function was significantly improved in both groups at 4, 8, and 12 weeks compared with baseline (p < 0.01) and the control group (p < 0.05). There was no significant difference in the incidence of severe asthma between the two groups (36.3 and 26.8%, respectively). Compared with the control group, the number of days and frequency of SABA use, as well as the awakenings at night were significantly reduced in the tiotropium group. There were no severe AE in either of the study groups.

Hamelmann and coauthors sought to assess the efficacy and safety of once-daily tiotropium Respimat in a phase III trial in adolescent patients with moderate symptomatic asthma [38]. In this 48-week, double-blind, placebo-controlled, parallel-group study, 398 patients aged 12–17 years were randomized to receive 5 or 2.5 mcg of once-daily tiotropium or placebo, administered through the Respimat device every evening, as add-on treatment to ICS background therapy with or without a leukotriene receptor antagonist; LABA therapy was not permitted during the trial. Improvement in peak FEV1 0–3 h at 24 weeks (primary end point) was statistically significant with both tiotropium doses compared with placebo: 5 mcg of tiotropium, 174 ml (95% CI 76–272 ml); 2.5 mcg of tiotropium, 134 ml (95% CI 34–234 ml). Significant improvements in trough FEV1 at week 24 (secondary end point) were also observed with the 5 mcg dose only. Trends for improvement in asthma control and health-related quality of life over the 48-week treatment period were observed. The overall incidence of AE, most mild or moderate in intensity, was comparable across the 3 treatment groups.

The same authors enrolled 392 adolescents, aged 12–17 years, with severe symptomatic asthma in a phase III double-blind parallel-group trial, to receive once-daily tiotropium 5, 2.5 µg, or placebo, as an add-on to ICS plus other controller therapies over 12 weeks [39]. The primary and key secondary end-points were change from baseline (response) in peak FEV1 0–3 h and trough FEV1 after 12 weeks of treatment. Tiotropium 5 and 2.5 µg provided numerical improvements in peak FEV1 0–3 h (90 ml, p = 0.104; 111 ml, p = 0.046, respectively) and trough FEV1 response, as well as in asthma control were observed with both tiotropium doses, compared with placebo. The safety and tolerability of tiotropium were similar with those of placebo.

A 12-week, phase III, double-blind, placebo-controlled, parallel-group trial sought to assess the efficacy and safety of tiotropium Respimat as add-on to background therapy in children with severe symptomatic asthma. Participants aged 6–11 years (n = 401) were randomized to receive once-daily tiotropium Respimat 5 mcg (2 puffs of 2.5 mcg) or 2.5 mcg (2 puffs of 1.25 mcg), or placebo [40]. Compared with placebo, tiotropium 5 mcg, but not 2.5 mcg, significantly improved the primary endpoint (peak FEV1 0–3 h) and the key secondary endpoint (trough FEV1). The safety and tolerability of tiotropium were comparable with those of placebo.

### Predictors of response

A total of 138 severe asthmatics with reduced lung function despite guideline recommended treatment were randomly assigned to additional tiotropium 18 mcg once a day and lung function parameters were measured every 4 weeks [41]. Responders were defined as those with an improvement of ≥ 15% or 200 ml in FEV1 that was maintained for at least 8 successive weeks. Single nucleotide polymorphisms (SNPs) in CHRM1–3 (coding muscarinic receptors M_1_–M_3_), as well as in the beta-2 adrenergic receptor (ADRB2) were recorded in 80 of the 138 asthmatics. Forty-six of the 138 asthmatics (33.3%) responded to tiotropium treatment. Logistic regression analyses (adjusted for age, gender, and smoking status) showed that ADRB2 Arg16Gly was significantly associated with a positive response to tiotropium.

The efficacy and safety of tiotropium compared to salmeterol and placebo in ADRB2 Arg16Arg adult patients with asthma not controlled by ICS alone was assessed in a double-blind, double-dummy, placebo-controlled trial [42]. After a 4-week run-in period with 50 mcg of twice-daily salmeterol, 388 asthmatic patients were randomized 1:1:1 to 16 weeks of treatment with 5 mcg of tiotropium Respimat administered once daily, 50 mcg of salmeterol administered twice daily through a metered-dose inhaler, or placebo. ICS regimens were maintained throughout the trial. Changes in weekly PEF from the last week of the run-in period to the last week of treatment (primary endpoint) were − 3.9 ± 4.87 l/min (n = 128) for tiotropium and − 3.2 ± 4.64 l/min (n = 134) for salmeterol, both significantly superior to placebo (− 24.6 ± 4.84 l/min, n = 125). Tiotropium was also non-inferior to salmeterol. AE were comparable across treatments.

More recently, a multisite, open-label, parallel-group, randomized clinical trial aimed to compare the effectiveness and safety of tiotropium vs LABA, when used with ICS in black adults with asthma and to determine whether allelic variation at the Arg16Gly locus of the ADRB2 gene was associated with treatment response [43]. It was in fact been reported that black populations may be disproportionately affected by LABA risks [44]. Patients eligible for, or receiving, step 3–4 combination therapy per National Heart Lung and Blood Institute (NHLBI) guidelines, were administered ICS plus either once-daily tiotropium (n = 532) or twice-daily LABA (n = 538) and were followed up for up to 18 months. Patients underwent genotyping at baseline and then attended study visits at 1, 6, 12 and 18 months, also completing monthly questionnaires. The primary outcome was time to asthma exacerbation. Secondary outcomes included patient-reported outcomes (Asthma Quality of Life Questionnaire, ACQ, Asthma Symptom Utility Index, and Asthma Symptom-Free Days questionnaire), FEV1, rescue medication use, asthma deteriorations, and AE. There was no difference between LABA + ICS vs tiotropium + ICS in time to first exacerbation, change in FEV1 at 12 and 18 months, ACQ score and other patient-reported outcomes at 18 months. Arg16Gly ADRB2 alleles were not associated with differences in the effects of tiotropium + ICS vs. LABA + ICS.

With the aim to describe individual and differential responses of asthmatic patients to salmeterol and tiotropium when added to ICS, as well as predictors of a positive clinical response, data from the double-blind, 3-way, crossover “NHLBI Asthma Clinical Research Network’s Tiotropium Bromide as an Alternative to Increased Inhaled Glucocorticoid in Patients Inadequately Controlled on a Lower Dose of Inhaled Corticosteroid trial” were analysed [45]. Although approximately equal numbers of patients showed a differential response to salmeterol and tiotropium in terms of morning PEF (n = 90 and n = 78, respectively) and asthma control days (n = 49 and n = 53, respectively), more showed a differential response to tiotropium for FEV1 (n = 104) than salmeterol (n = 62). An acute response to SABA (i.e. albuterol) significantly predicted a positive clinical response to tiotropium for FEV1 and morning PEF, as did a decreased FEV1/FVC. Higher cholinergic tone was also a predictor, whereas ethnicity, sex, atopy, IgE level, sputum eosinophil count, fraction exhaled nitric oxide, asthma duration, and body mass index were not.

Additionally, to determine whether the efficacy of tiotropium add-on therapy is dependent on patients’ baseline characteristics two randomized, double-blind, parallel-group, twin trials of once-daily tiotropium Respimat 5 mcg add-on to ICS plus LABA were performed in parallel in patients with severe symptomatic asthma [46]. Exploratory subgroup analyses were performed to determine whether results were influenced by baseline characteristics. Patients were randomized to receive tiotropium (n = 456) or placebo (n = 456). Tiotropium improved lung function, reduced the risk of asthma exacerbations and asthma worsening, and improved asthma symptom control, compared with placebo, independently from baseline characteristics including gender, age, body mass index, disease duration, age at asthma onset, FEV1% predicted and reversibility.

### Non-bronchodilator effects

Although the use of tiotropium in asthma has been long advocated to exert its activity on airway tone, alternative mechanisms have been demonstrated in in vitro studies and in animal models to explain the efficacy of LAMA. Indeed, since the different cells involved in the inflammatory cascade express muscarinic receptors, it is plausible to hypothesize that tiotropium can act by interfering (or modulating) the function of these cells. These ancillary effects of tiotropium could corroborate the bronchodilator effect of the drug. Given the potential relevance of these findings for asthmatic patients, in addition to the evidence retrieved through the search strategy, the principal data on non-bronchodilator effects of tiotropium are summarized below.

The influx of eosinophils and neutrophils in the airways of asthmatics is in part modulated by M_3R_ activation [47]. Tiotropium has been found to cause a reduction in the eosinophil deposition in the airways [48], by acting directly on M_3R_ present on eosinophils or through the blocking of non-neuronal ACh release from macrophages and epithelial cells. Bühling and coauthors showed in vitro a suppressive activity of tiotropium on the ACh-mediated production of macrophages-derived chemotactic mediators [49]. Eosinophilic recruitment was shown to be affected also by a concomitant administration of tiotropium and budesonide or ciclesonide in animal models [50, 51]. In conjunction with olodaterol, tiotropium showed a synergistic protective effect against allergen-induced hyper-responsiveness in guinea pig models of asthma, inhibiting both the early and late asthmatic phases [52].

Even more important, M_3R_ seem to mediate airway smooth muscle thickening and extracellular matrix deposition. Profita et al. demonstrated an increased expression of M_1R_ and M_3R_ in fibroblasts from COPD and smoker subjects compared with those from healthy subjects [53]. After exposure to ACh, the proliferation of fibroblasts increased, and this phenomenon was down-regulated by tiotropium and mediated by M_R_, extracellular signal-regulated kinase (ERK) 1/2 and nuclear factor (NF) kappaB. The ACh-induced proliferation of fibroblasts was shown to be inhibited by tiotropium in a dose–response fashion [54]. The anti-remodelling effect of tiotropium was also confirmed by studies in animal models. In mice chronic asthma models tiotropium significantly decreased smooth muscle thickening and peribronchial collagen deposition, with a parallel reduction of Th2-mediated cytokines such as IL5 and IL13 [55].

An additional mechanism through which an anticholinergic drug may exert its beneficial effects in chronic airway diseases is by modulating mucus production. MUC5AC is recognized as the most common mucin gene whose expression is markedly increased in asthma and is mediated by M_3R_ in goblet cells [56]. MUC5AC production from goblet cells in mice was found to be inhibited by selective M_3R_ with tiotropium [57]. The effect of tiotropium on mucus production is not accompanied by modification of the rheological properties of mucus.

Finally, since cough is an important symptoms of asthma, and a cough-variant asthma has been described in clinical practice, the effect of LAMAs on cough reflex have been explored. Birrell and colleagues showed that tiotropium, but not glycopyrronium, was able to modulate the cough reflex through the transient-potential vanilloid receptor type-1 (TRPV1) with mechanisms that have not been fully understood, and perhaps not related to the intrinsic anticholinergic activity [58]. In this respect, Mutolo and coworkers recently demonstrated that the beneficial effect of tiotropium on cough involves acid-sensing ion channels and mechanoreceptors [59].

### Cost effectiveness

The cost effectiveness of tiotropium therapy as add-on to usual care in asthma patients that are uncontrolled despite treatment with ICS/LABA combination was assessed from the perspective of the UK National Health Service (NHS). A Markov modelled analysis of two clinical trials, developed to determine levels of asthma control and exacerbations, found that add-on tiotropium provided an incremental 0.19 QALYs and £5389 costs over a lifetime horizon, resulting in an incremental cost-effectiveness ratio of £28,383 per QALY gained [60, 61].

### Safety

Patients with symptomatic asthma (n = 285), despite treatment with ICS-LABA, were randomised to once-daily tiotropium (5 and 2.5 μg) Respimat for 1 year in a double-blind, placebo-controlled, parallel-group study to evaluate its long-term safety profile [62]. Secondary endpoints included trough FEV1, PEF response and ACQ-7 score. At week 52, AE rates with tiotropium 5, 2.5 μg and placebo were 88.6, 86.8 and 89.5%, respectively. Most commonly reported AE were pharyngitis, nasopharyngitis, asthma, bronchitis and gastroenteritis. In the tiotropium 5, 2.5 μg and placebo groups, 8.8, 5.3 and 5.3% of patients reported drug-related AE; 3.5, 3.5 and 15.8% reported serious AE. At week 52, adjusted mean trough FEV1 and PEF responses were significantly higher with tiotropium 5 μg (but not 2.5 μg) versus placebo. ACQ-7 responder rates were higher with tiotropium 5 and 2.5 μg versus placebo at week 24. Authors concluded that the long-term tiotropium Respimat safety profile was comparable with that of placebo, and associated mainly with mild to moderate AE in patients with symptomatic asthma despite ICS-LABA therapy.

## Discussion

Collected findings strongly support a significant beneficial effect of tiotropium on several lung function parameters in both the adult and paediatric asthma populations. Retrieved evidence is of high quality being supported by studies with a robust design (i.e. randomized, double blind clinical trials), performed in large samples and published in top-ranked scientific journals. Most of the data addressed the role of tiotropium delivered by the Respimat soft-mist inhaler, showing best results for the once daily 5 mcg dose. Despite only one trial has been designed and powered for safety as primary outcome (prompting further ad-hoc investigations), the drug has been consistently shown a harmless profile when compared to placebo or other active treatments. Tiotropim has been proven to be effective irrespective of demographic patient characteristics (i.e. gender, age, body mass index, disease duration, age at asthma onset). The studies reporting scores from the Asthma Quality of Life Questionnaire (AQLQ) substantially failed in showing a significant and clinical relevant benefit of tiotropium over LABA/ICS alone despite the effect estimate favoured add-on tiotropium [63]. Conflicting data exist regarding a possible role of ADRB2 single nucleotide polymorphisms in predicting a positive drug response. This aspect might be of high clinical relevance and should deserve further assessment. The efficacy and safety of inhaled LABA in asthmatic patients with the ADRB2 Arg16Arg genotype has been in fact questioned, and the use of antimuscarinic agents may be proposed as an alternative treatment strategy in patients whose symptoms are not controlled by ICS.

In conclusion, the recent positioning of tiotropium as an additional treatment in some forms of asthma is the result of a growing evidence of its efficacy. The anticholinergic effect provides a rationale for the appropriate management of increased bronchomotor tone, which is paramount in chronic airway diseases. The renewing attention to the role of LAMA in asthma has led to the exploration of new pathways. It is plausible to hypothesize that the potent effect of tiotropium is to be attributed to mechanisms other than simply reducing the smooth muscle tone. The observations of anti-inflammatory properties or reduction in mucus production are interesting but at present demonstrated in in vitro studies and in animal models, and advocate for further, specifically designed investigations. Whether the efficacy of tiotropium is a class effect or, as suggested by several studies, a peculiar aspect of the drug is yet to be determined. The research developed and the ongoing/future studies on the demonstration of efficacy and safety of tiotropium in asthma represent an extremely valuable contribution for its optimal management.
